# Environment and Reproductive Health in China: Challenges and Opportunities

**DOI:** 10.1289/ehp.1205117

**Published:** 2012-05-01

**Authors:** Weihua Li, Bo Chen, Xuncheng Ding

**Affiliations:** Shanghai Institute of Planned Parenthood Research, Shanghai, China, E-mail: iamliweihua@yahoo.com; School of Public Health, Fudan University, Shanghai, China; Shanghai Institute of Planned Parenthood Research, Shanghai, China

Although maternal and infant mortality has been reduced in China over the past several years, the country is still facing big challenges in reproductive health (Fang and Kaufmann 2008). In 2001, the prevalence of birth defects reported in China was higher than that in most developed countries (Christianson et al. 2006), and the prevalence increased from 8.8 per thousand in 1996 to 15.0 per thousand in 2010 [Ministry of Health, People’s Republic of China (MOH) 2011]. According to a report of the National Population and Family Planning Commission of China (Zhou et al. 2011), infertility also increased—from a national incidence of 6.9% in 1976–1985 to 17.1% in 2001.

One possible contributor to the reproductive health burden in China may be environmental pollution. The most populous country in the world, China has under-gone rapid economic develop-ment during the past 30 years. Nevertheless, this success also increases energy use and industrial waste, resulting in severely polluted air and water. Air quality in China is among the worst in the world, with air pollution levels in many cities well above permissible limits, and half of the water resources in China are considered too polluted for human use (Zhang et al. 2010a). Many industrial chemicals that have been released into the environment are endocrine disruptors that are associated with adverse effects on human reproductive health (Balabanic et al. 2011).

Surveys in China have shown associations between environmental pollution and reproductive health, and specific environ-mental pollutants have been correlated with increased prevalence of reproductive damage. For example, polychlorinated biphenyls (PCBs) were used mainly in eastern and central China; in a survey of birth defects in China, Zhu (2008) found that the prevalence of hypospadias declined gradually from east to west. In another study, Wang et al. (2008) investigated effects of pollution in the Black River in Henan Province, a river that flows through five counties and picks up > 90% of the local industrial waste-water and domestic sewage. These authors found that the incidence of spontaneous abortion, malformations, low birth weight, and stillbirth was significantly higher for fertile women who lived by the river than for those who lived 5–10 km away from the river. In a cohort study in Tongliang county conducted in 2002–2007, Perera et al. (2008) found that after the shutdown of a nearby coal-fired power plant, the level of polycyclic aromatic hydro-carbons (PAHs) generated by the plant was reduced and children’s neuro-behavioral develop-ment was significantly improved.

In China, the nation-wide prevalence of birth defects in the last two decades has undergone the same increasing trend as environ-mental pollution. Areas in western China have lower prevalence of birth defects than do the coastal areas, where pollution is generally more severe (MOH 2011). From 1996 to 2009, the prevalence of birth defects rose 105.5% in urban areas, compared with 21.7% in rural areas (MOH 2011). These values suggest that possible chemi-cal exposure in association with birth defects should be investigated. For example, the prevalence of neural tube defects (NTDs) in Shan’xi Province is among the highest in China, and spatial analyses revealed an association between production in coal mines and prevalence of NTDs in coal-mining areas (Liao et al. 2010). Also in Shan’xi Province, one case–control study found that the prevalence of NTDs was associated with indoor air pollution from coal combustion (Li Z et al. 2011), and another study reported that the prevalence of NTDs was related to the elevated placental concentrations of PAHs, *o,p*-dichloro-diphenyl-trichloro-ethane (*o,p*-DDT), and α-hexa-chloro-cyclo-hexane (Ren et al. 2011).

Another challenge is infertility. Environmental endocrine disruptors (EEDs), including pesticides, heavy metals, phthalates, and PAHs, are suspected risk factors for infertility (Balabanic et al. 2011). Declining semen quality parameters (semen concentration and sperm motility and morphology) have been associated with environ-mental exposure to bisphenol A (Li DK et al. 2011), dis-infec-tion by-products (Xie et al. 2011), and pyrethroids (Ji et al. 2011). In an analysis of fertility data from 11,726 fertile Chinese males, Zhang et al. (1999) found that sperm counts decreased from 103.0 × 10^6^ in 1981 to 83.8 × 10^6^ in 1996, and the percentage of sperm with normal morphology decreased from 85.0% to 77.9% during the same period. These changes in semen quality could be related to long-term exposure to EEDs.

Child growth and development are also serious health concerns in China. In a national survey by the Chinese Medical Association during 2003–2005, 19.6% of 20,654 girls had evidence of breast develop-ment at 8 years of age (among the earliest recorded worldwide), and the median age of menses was 12.27 years—1.23 years earlier than in 1979 (Ma et al. 2009). Earlier onset of puberty has been associated with certain EEDs in both animal and human studies (Mouritsen et al. 2010).

Childhood and adolescent overweight and obesity are also becoming problems in China. Data from the China Health and Nutrition Survey showed that the prevalence of over-weight for school-children and adolescents doubled from 1991 to 2004, increasing from 6.5% to 16.1% in children 6–11 years of age and from 3.3% to 6.2% in adolescents 12–18 years of age (Zhang et al. 2010b). In epidemiological studies, body mass index (BMI) in adults and children has been associated with exposure to EEDs such as dichloro-diphenyl-dichloro-ethylene (DDE), PCBs, and phthalates (Hatch et al. 2010).

The annual gross domestic product (GDP) in China is predicted to quadruple by 2020, which could pose more serious environmental challenges to the country. Because of China’s high levels of environmental pollutants as a result of industrialization, reproductive health risks need to be assessed. There is a lack of comprehensive and persuasive reproductive health studies in China; thus, large epidemiological studies—especially prospective cohort studies— are needed to examine populations in highly polluted areas.

China is taking more aggressive steps to support reproductive health research. Its unique family planning service network, established in 1978, has become a comprehensive vertical system for reaching villages and communities, which can be used in epidemiological studies. For example, China’s “Birth Defect Intervention Project,” which is based on this network, was launched by the National Population and Family Planning Commission of China in 1999 and is aimed at covering all cities nationwide. Furthermore, reproductive health research was listed as one of the priority items in China’s Long-term Science and Technology Development Program Outline for 2006–2020 (State Council of the People’s Republic of China 2006). Even though China has all of these programs in place, an information network is urgently needed to enable sharing of new data, especially between environ-mental protection and public health agencies, so integrated and updated information will be available across the sectors.
